# Efficacy of Topical Liposomal Amphotericin B versus Intralesional Meglumine Antimoniate (Glucantime) in the Treatment of Cutaneous Leishmaniasis

**DOI:** 10.1155/2011/656523

**Published:** 2011-11-24

**Authors:** Pouran Layegh, Omid Rajabi, Mahmoud Reza Jafari, Parisa Emamgholi Tabar Malekshah, Toktam Moghiman, Hami Ashraf, Roshanak Salari

**Affiliations:** ^1^Research Center for Skin Diseases & Cutaneous Leishmaniasis, Ghaem Hospital, Faculty of Medicine, Mashhad University of Medical Sciences, Mashhad, Iran; ^2^Department of Medicinal Chemistry, Mashhad University of Medical Sciences, Mashhad, Iran; ^3^Department of Pharmaceutics, Mashhad University of Medical Sciences, Mashhad, Iran; ^4^Department of Dermatology, Ghaem Hospital, Mashhad University of Medical Sciences, Mashhad, Iran; ^5^Mashhad University of Medical Sciences, Mashhad, Iran; ^6^Buali (Avicenna) Research Institute, Mashhad University of Medical Sciences, Mashhad, Iran

## Abstract

*Background*. Topical treatment of cutaneous leishmaniasis is an attractive alternative avoiding toxicities of parenteral therapy while being administered through a simple painless route. Recently liposomal formulations of amphotericin B have been increasingly used in the treatment of several types of leishmaniasis. *Aims*. The efficacy of a topical liposomal amphotericin B formulation was compared with intralesional glucantime in the treatment of cutaneous leishmaniasis. *Methods*. From 110 patients, the randomly selected 50 received a topical liposomal formulation of amphotericin B into each lesion, 3–7 drops twice daily, according to the lesion's size and for 8 weeks. The other group of 60 patients received intralesional glucantime injection of 1-2 mL once a week for the same period. The clinical responses and side effects of both groups were evaluated weekly during the treatment course. *Results*. Per-protocol analysis showed no statistically significant difference between the two groups (*P* = 0.317, 95% confidence interval (CI) = 1.610 (0.632–4.101)). Moreover, after intention-to-treat analysis, the same results were seen (*P* = 0.650, 95% CI = 0.1.91 (0.560–2.530)). Serious post treatment side effects were not observed in either group. *Conclusions*. Topical liposomal amphotericin B has the same efficacy as intralesional glucantime in the treatment of cutaneous leishmaniasis.

## 1. Introduction

Leishmaniasis is a major world health problem, which is increasing in incidence. The diagnosis is often made on the basis of a clinically typical lesion in conjunction with an appropriate history of exposure [1]. Although cutaneous leishmaniasis (CL) is a spontaneously resolving disease usually within a year, the resulting disfigurement and the duration of the disease require an effective treatment [2]. A wide range of therapeutic options have been employed over the years but its optimal treatment is not yet known whereas drug resistance is becoming an increasing problem in countries of leishmaniasis endemicity [3]. 

Amphotericin B (AmB), a polyene antibiotic and the gold standard for systemic fungal infections, also has excellent antileishmanial activity. Due to the higher affinity of AmB for 24-substituted sterols, aqueous pores are formed in the membrane leading to increased membrane permeability and killing of *Leishmania *[4]. It is commonly administered intravenously as an alternative treatment in visceral and mucocutaneous leishmaniasis [5–7]. In a mouse model it has been shown that topical AmB as a complex either with cholesteryl sulfate or with phospholipids in the presence of ethanol can penetrate into the skin and cure CL in a localized manner using a very low total drug concentration [8]. In another study topical AmB was proven to be effective against CL caused by *Leishmania major *[9]. Since liposomal AmB is administered topically, it could be considered as a good alternative in the treatment of CL.

In the present study we aim to compare the therapeutic effects of a formulation of topical liposomal AmB with intralesional glucantime in the treatment of CL.

## 2. Material and Methods

This clinical trial was carried out on CL patients who visited the dermatology clinic from March 2008 through September 2010 in Ghaem Hospital, Mashhad, Iran, where CL is endemic and 96.5% of CL cases are caused by *L. tropica *and less commonly *L. major *[10, 11]. 110 patients with the clinical diagnosis of CL were recruited and were completely informed of the study goals and had filled in the consent required to participate in the trial, which was also approved by the ethics committee of Mashhad University of Medical Sciences.

The inclusion criteria were

- patients with cutaneous leishmaniasis approved by either a direct smear stained with Giemsa or a positive skin biopsy of lesions with less than 6-month duration,
- in cases with a previous history of anti-leishmaniasis therapy, a 3-month treatment-free interval from the last treatment course was taken into consideration.

The exclusion criteria were

- pregnancy,
- breastfeeding,
- taking any other specific treatment while participating in the study,
- past medical history of any local or systemic disease during the last 2 months,
- a significant underlying disease such as cardiac, renal, or liver dysfunction.

The information collected from each patient included demographic data and characteristics of their disease. 

 The 110 patients who met our inclusion criteria were randomly divided into two groups. Liposomal AmB was administered for 50 patients while the other 60 received intralesional glucantime.

 In the first group liposomal AmB was administered as 3–7 drops twice daily according to the lesion size. In the second group intralesional glucantime (Glucantime; Specia, Paris, France) was injected into each lesion once a week, to the point when the lesion's surface became fully infiltrated and up to a maximum dose of 2 mL.

Clinical response was determined on the basis of the lesion induration size of and the extent of re-epithelialization in ulcerative ones on every follow-up visit. The treatment period was decided as 8 weeks for each group and the patients were followed up weekly during the treatment course while the changes in the lesion induration size and side effects were recorded in every session. The patients were also studied once again, 6 months after termination of the treatment course. The therapeutic results were classified as follows:

- slight improvement: decrease in induration size up to 25%,
- mild improvement: decrease in induration size between 25 and 50%,
- moderate improvement: decrease in induration size between 50 and 75%,
- marked improvement: decrease in induration size more than 75%.

### 2.1. Materials

Egg lecithin, cholesterol, glucose, oleic acid and AmB (formulated with deoxycholate) (250 *μ*g/mL) were purchased from Sigma-Aldrich.

### 2.2. Equipments

The equipments employed included a transmission electronic microscope (LEO 912 AB, Germany), freeze drier (Labcon Co., USA), Spectrophotometer (UV-160A Shimatzu, Japan) and a Particle size analyzer (PSA) (Klots, Germany).

Multilamellar large vesicle (MLV) liposomes containing AmB were prepared through the freeze-drying method.

### 2.3. Method

The method was based on the formation of a homogenous dispersion of lipids in water-soluble carrier materials. To obtain the lipid-containing solid dispersion, liposome-forming lipids and water-soluble carrier materials with AmB were dissolved in t-Butyl alcohol/water cosolvent systems to form an isotropic monophase solution, and then the resulting solution was frozen and lyophilized after sterilization by filtration through 0.2 *μ*m pores. Adding water to the lyophilized product besides hard shaking followed by sonication spontaneously forms a homogenous liposome preparation.

For the formation of monophase solution and sterile filtration, egg lecithin (375 mg), cholesterol (125 mg), and oleic acid (25 mg) were dissolved in t-Butyl alcohol, while glucose (500 mg) and AmB were dissolved in water. Then these two solutions were mixed together in appropriate ratios (1 : 1) to give a third clear isotropic monophase solution. After the monophase solution was sterilized by filtration through 0.22 *μ*m pores, it was filled into the 100 mL freeze-drying flask vials with a fill volume of 10.0 mL [12]. 

### 2.4. Freeze-Drying

The freeze-drying process was as follows: (1) freezing at −40°C for 8 hrs; (2) primary drying at −40°C for 24 hrs; (3) secondary drying at 25°C for 10 hrs. The chamber pressure was maintained at 20 Pascals during the drying process. Note that, lyophilized products can be stored for a long period of time under the dry ambient providing it is well sealed.

**Reconstitution**

Lyophilized products can be reconstituted by adding 10 mL of AmB solution (250 *μ*g/mL) and gentle sterile water with hard shaking followed by sonication, which will lead to the formation of the aqueous suspensions of homogeneous liposomes.

### 2.5. Results

The encapsulation capacity of the liposome was found to be 81.4 ± 3.12% by the UV spectroscopic method, indirectly. The average particle size, which was measured by PSA was 2.21 *μ*m (PdI = 0.254).

### 2.6. Safety Measurements

The possible transdermal passages of AmB into blood circulation were previously ruled out [9]. The apparent volume of distribution of liposomal form of AmB is reported to be 0.1–0.44 lit/kg while the maximum therapeutic concentration of this drug is about 1–1.5 mg/kg [13]. Liposomal formulation of AmB contains 5 mg of active ingredient per 1 mL of solution; supposing each drop volume (0.05 mL) involved 0.25 mg amphotericin, the amount of the drug in 2 drops (one dose of treatment) would be equivalent to 0.5 mg of active ingredient.

Considering the apparent volume of distribution [14], the drug concentration in the plasma of a 70 kg person will be between 0.016 and 0.07 mg/lit, which is less than the maximum therapeutic dose for this drug (2.26–10 mg/lit).

### 2.7. Sample Size

To detect a 35% difference in the cure rate between the two groups of liposomal AmB and glucantime, assuming a 90% cure rate in liposomal AmB group with a 95% power and a 5% two-sided type I error, 36 subjects were required in each group. Also regarding a loss to follow up of 20%, this number raised to 50 patients for each group.

### 2.8. Statistical Analysis

Two different approaches were performed to compare the treatment outcomes between the two groups. The first approach was an intention-to-treat analysis that included all the 110 patients enrolled at the beginning of the trial and considered patients with irregular treatments and followups as therapeutic failures. The second was more stringent and solely included the 76 patients with regular treatments and followups. They were considered as good compliers and better represented an explanatory approach which allowed the interpretation of data in terms of effectively treated patients.

Statistical analysis was done by applying Chi-square test. Data was expressed as mean values ± SD, and the difference was considered significant when *P* < 0.05.

## 3. Results

**Figure 1:**
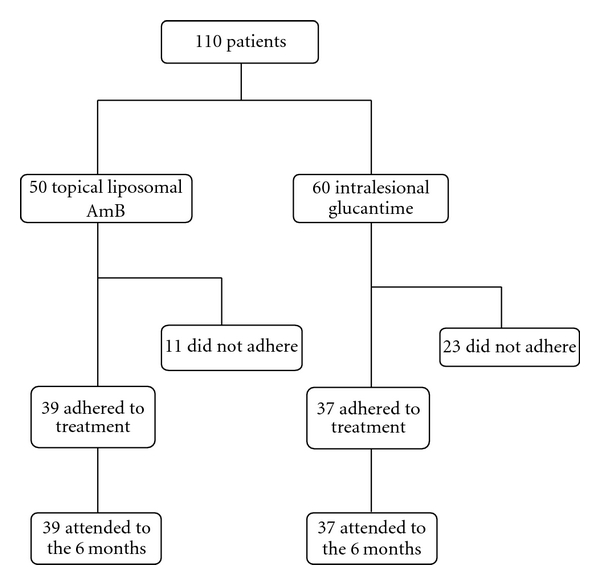
The flow of participants through each stage of our study.

Figure 1 shows the flow of participants in our study. During the study, 11 patients in the liposomal AmB and 23 in the glucantime groups were excluded due to taking two treatment methods simultaneously, not completing the treatment course, changing their home, and losing access for further followup while 76 completed the study including 39 in the study group (liposomal AmB) and 37 in the control group (intralesional glucantime). Demographic characteristics of the patients in both groups have been shown in Table 1.

According to these data, the two groups were matched for sex, age, number of lesions, lesion duration, type, and location (*P* > 0.05) but varied in the type and number of lesions which was a slightly higher number of papule and plaque lesions in the glucantime group.

The most common lesion site in all the studied cases was the upper limbs (hand and forearm: 45.4%) and the head and neck (face: 43.6%), respectively, while the most common lesion type in both groups was papule and plaque (78.2%).

**Figure 2:**
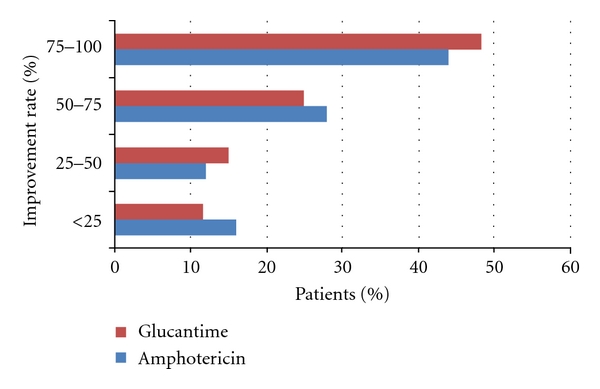
Improvement rate of the two groups based on change in induration size of lesions.

 No significant difference in the treatment response between the males and females was seen in either group (*P* = 0.840). Figure 2 compares the improvement rate of both groups based on changes in induration size, and Table 2 summarizes the results of the intention-to-treat approach and the analysis of “compliers only,” comparing therapeutic failure rates. Serious posttreatment side effects were not observed in either group. 7 patients (11.7%) in the glucantime group showed erythema and edema at the injection site which were managed with a cold compress and antihistamine drugs and they all completed the treatment course. In the latter group only 1 patient (1.7%) complained of hypersensitivity. Also 5 patients (10%) in the AmB group showed mild pruritus around the lesions. Upon 6 months of follow-up, no recurrence of the disease was noted in the cured patients of either group.

## 4. Discussion

Mashhad located in the Northeast of Iran is endemic for cutaneous leishmaniasis mainly caused by *L. tropica* and less commonly *L. major *[10, 11, 15]. Pentavalent antimoniates have long been the most common treatment for leishmaniasis in our region, but acquired drug resistance towards them has increased during the recent years [16, 17], raising a strong need for new anti-leishmaniasis treatments. Knowing the fact that CL is a self-limiting disease, the main goal in its treatment would be controlling the spread of the disease in endemic regions besides decreasing scar formation; therefore it would be more logical to use safe topical drugs in order to prevent any adverse reactions. Systemic liposomal AmB has been recently administered in the treatment of drug-resistant cutaneous leishmaniasis [18–20]. Preliminary results have shown that topically applied lipid-based formulations containing AmB have a significant therapeutic effect in the treatment of CL lesion in adults. Zvulunov et al. even reported successful treatment of a 1.5-year-old infant with persistent CL by the administration of a topical colloidal solution of AmB for 3 weeks [21]. In a study conducted in 1999 on the treatment outcome of two similar lesions of the same individual, the topical liposomal AmB-treated lesions showed significant improvement with no evidence of relapse when compared to the placebo-treated ones [9]. Another study taking place in Israel on 19 patients receiving 2 vials, one containing AmB-cholesteryl sulfate dispersed in a 5% ethanol solution and one with 5% ethanol in water for half of each patient's lesions, the results revealed a better therapeutic response in those lesions treated with topical liposomal AmB in comparison to placebo [22]. 

Although several studies have been performed on the efficacy of topical AmB on CL, this study is the first which compares its effect with intralesional glucantime; moreover, sample size of our study is not comparable with other similar ones.

In the present study, comparing the effect of AmB and intralesional glucantime on leishmania lesions, no significant difference was observed (56.4% versus 67.6%, *P* = 0.317), indicating that topical AmB has a similar efficacy to intralesional glucantime. Although preparing topical liposomal AmB as a therapeutic drug is neither easy nor inexpensive, because of its simple, painless administration route by the patient himself, no transdermic passage into blood circulation causing less toxicity, and no need for recurrent visits, AmB could be considered as an alternative treatment especially in children who do not tolerate painful methods, patients who cannot make recurrent visits, and in cases where glucantime is contraindicated or unresponsive.

Evaluating the effect of a drug on a self-limiting disease, in particular, considering the fact that variability in the disease length in different individuals can be of several months, would be quite difficult; therefore a study in which patients with two similar leishmania lesions could simultaneously receive both treatments (AmB and glucantime) is highly recommended in further studies on this issue.

## Figures and Tables

**Table 1:** Demographic and cutaneous leishmaniasis characteristics of the studied population.

	Intralesional glucantime	Liposomal amphotericin B	*P *value
No. of patients	60	50	
*Sex *			
Male	21	23	0.241
Female	39	27	
*Age *			
(mean ± SD)	25.30 ± 15.70	20.54 ± 18.72	0.150
No. of lesions	1.4 ± 0.76	1.91 ± 1.02	<0.05
(mean ± SD)			
*Duration of lesions (week)*	3.84 ± 1.75	4.24 ± 1.24	0.166
*Type of lesions *			
Papuloplaque	53	33	0.011
Nodule	5	8	
Ulcer	2	9	
*Location of lesions *			
Head and neck	22	26	0.185
Hand	32	18	
Leg and trunk	6	6	

**Table 2:** Comparison of therapeutic response rates in the studied groups using the intention-to-treat approach and per-protocol analysis.

Proportion (%) of patients with cure at 8 weeks				
Analytical assumption^a^	AmB group	GL group	OR (95% CI)	*P*
			(AmB versus GL)	
Per protocol	22/39 (56.4)	25/37 (67.6)	1.610 (0.632, 4.101)	0.317
ITT	22/50 (44.0)	29/60 (48.3)	1.91 (0.560, 2.530)	0.650

GL: glucantime; AmB: liposomal amphotericin B; ITT: intention-to-treat; OR: odds ratio; CI: confidence interval.

^a^Per protocol: analysis excluding patients that were lost to follow up, ITT: analysis including patients lost to follow up throughout the study, who were considered to have experienced treatment failure.
